# The Epidemiological Situation of the Managed Honey Bee (*Apis mellifera*) Colonies in the Italian Region Emilia-Romagna

**DOI:** 10.3390/vetsci9080437

**Published:** 2022-08-17

**Authors:** Giovanni Cilia, Elena Tafi, Laura Zavatta, Valeria Caringi, Antonio Nanetti

**Affiliations:** CREA Research Centre for Agriculture and Environment, Via di Corticella 133, 40128 Bologna, Italy

**Keywords:** health status, DWV, ABPV, CBPV, KBV, *Nosema ceranae*, trypanosomatids, diseases, pathogens, monitoring

## Abstract

**Simple Summary:**

The collapse of honey bee colonies is an important phenomenon worldwide. The individual and synergic actions of pathogens are one of the causes of this decline. Monitoring programs are essential to understand and prevent the epidemiological patterns that are involved. The present study aimed to investigate the health status of honey bees in the Emilia–Romagna region (northern Italy) during the year 2021, on workers from 31 apiaries. The prevalence and abundance of DWV, KBV, ABPV, CBPV, *Nosema ceranae*, and trypanosomatids (*Lotmaria passim, Crithidia mellificae, Crithidia bombi*) were investigated four times in the year using molecular methods. Trypanosomatids were not found in any of the samples, while DWV, CBPV and *N. ceranae* were the most prevalent pathogens. Pathogens had different peaks in abundance over the months, showing seasonal trends related to the dynamics of both bee colonies and *Varroa destructor* infestation. The results of this study suggest that the monitoring program could be useful to understand the dynamics of honey bee pathogens.

**Abstract:**

The recent decades witnessed the collapse of honey bee colonies at a global level. The major drivers of this collapse include both individual and synergic pathogen actions, threatening the colonies’ survival. The need to define the epidemiological pattern of the pathogens that are involved has led to the establishment of monitoring programs in many countries, Italy included. In this framework, the health status of managed honey bees in the Emilia–Romagna region (northern Italy) was assessed, throughout the year 2021, on workers from 31 apiaries to investigate the presence of major known and emerging honey bee pathogens. The prevalence and abundance of DWV, KBV, ABPV, CBPV, *Nosema ceranae*, and trypanosomatids (*Lotmaria passim, Crithidia mellificae, Crithidia bombi*) were assessed by molecular methods. The most prevalent pathogen was DWV, followed by CBPV and *N. ceranae.* Trypanosomatids were not found in any of the samples. Pathogens had different peaks in abundance over the months, showing seasonal trends that were related to the dynamics of both bee colonies and *Varroa destructor* infestation. For some of the pathogens, a weak but significant correlation was observed between abundance and geographical longitude. The information obtained in this study increases our understanding of the epidemiological situation of bee colonies in Emilia–Romagna and helps us to implement better disease prevention and improved territorial management of honey bee health.

## 1. Introduction

The health status of managed honey bees (*Apis mellifera* L.) is threatened by several pests and pathogens [1]. The effects of these infections and their interactions with other biotic (e.g., bee genetics, queen behaviour) and abiotic (e.g., climate changes, lack of forage, exposition to agrochemicals, and management practices) factors can lead to colony losses [2,3,4]. Although both the symptoms and effects caused by contact with pesticides have been investigated [3,4], the role of pathogens and their effects in relation to their abundance, co-infection and seasonality need to be clarified [5]. Both disease occurrence and fatal events such colony losses vary widely among countries and climatic regions [1,6,7]. Usually, colony losses occur in the wintertime or the early spring, but they may also occur in summer and autumn, as the result of incorrect beekeeping management and/or pathogens [8,9]. Clarity regarding the epidemiological diffusion of bee pathogens could be helpful to understand and/or prevent any sanitary problems.

Often, honey bee pathogens are globally distributed. They include bacteria, fungi, microsporidia, viruses, trypanosomatids, and mites [10,11], which may act individually or synergically [5,12,13,14]. Under specific conditions, their detrimental effect may induce Colony Collapse Disorder (CCD) [15,16]. In this context, *Nosema ceranae* infection and viral diseases are considered to play a pivotal role [11].

*N. ceranae* is an obligate intracellular microsporidian, which is causing nosemosis type C in western honey bees [17,18,19] due to the infection of the ventricular epithelial cells [20,21]. This pathogen is spread globally and affects honey bees at both individual and colony levels, inducing lethargic behaviour in worker bees, reducing lifespan, and leading to poor honey and pollen harvest [22,23,24,25].

Deformed wing virus (DWV) is a non-enveloped ssRNA (+) virus belonging to *the Iflavirus* genus [26]. This virus is the most studied and the most prevalent honey bee pathogen [27,28]. DWV is often associated with the *Varroa destructor* infestations, as the mite may transmit the virus through trophic activity [29,30]. Symptomatic DWV infections result in adults with anatomical deformities such as crippled wings and shortened abdomens, although asymptomatic courses are frequent and cause colony dwindling and collapse [30,31,32].

Acute bee paralysis virus (ABPV) and Kashmir bee virus (KBV) are two non-enveloped ssRNA (+) viruses within the *Dicistroviridae* family belonging, respectively, to the *Apavirus* genus and *Cripavirus* genus [28,33,34]. These viruses are genetically similar and, together with Israeli acute paralysis virus (IAPV), are considered to form a complex (ABPV-KBV-IAPV complex) [35,36]. Both viruses could be horizontally transmitted by *V. destructor* [35,37]. Although ABPV and KBV are highly virulent, their spread in the hive is limited. They infect brood and adults asymptomatically at both individual and colony levels, although ABPV-infected workers may rapidly evolve paralysis [35].

Chronic bee paralysis virus (CBPV) is an unclassified enveloped ssRNA (+) virus, which is unusual as its genome consists of two single- or plus-stranded RNAs [38,39]. The virus causes a complex disease in the workers, consisting of ataxia, and the inability to fly, as a result of its neurotropism [38,40]. Symptomatic honey bees are unable to fly and show a dark, hairless, and trembling abdomen [38,41]. Although the disease was once known to be highly seasonal and related to the spring development of the colonies, recent reports indicate its presence throughout the year and, consequently, its increased prevalence [42].

*Lotmaria passim*, *Crithidia mellificae*, and *C. bombi* are three trypanosomatids capable of infecting the bee’s gastrointestinal tract [43,44]. *L. passim* and *C. mellificae* are two pathogens that are frequently detected in *A. mellifera* [44,45,46]. *C. bombi*, although first isolated in bumblebees, is also known to infect honey bees [47], which act as vector of the trypanosomatids [48]. They all spread oro-faecally among colony members [48], and infection could alter bee behaviour and reduce their lifespan [49,50,51,52,53]. Among the three species, *L. passim* is considered to have the largest global distribution and, as such, it has replaced previously misidentified *C. mellificae* infections [44].

All the above-reported pathogens are present in Italy; nonetheless, scant information is available for KBV and trypanosomatids. KBV was found in the years 2013 and 2017 in one single apiary of the Latium region [54] and *Vespa velutina* individuals, respectively [55]. *L. passim* was reported as present in the Veneto region only [56].

This study was conducted to contribute to our understanding of the epidemiological situation of honey bee diseases in Italy. Samples from 31 apiaries located in the Emilia-Romagna region were collected and analyzed in the year 2021, to quantify both the prevalence and abundance of *N. ceranae*, DWV, KBV, ABPV, CBPV, *L. passim*, *C. mellificae*, and *C. bombi*. These pathogens were selected for the following reasons: (i) they are not responsible for notifiable diseases in Italy, (ii) molecular methods are available for their assessment, (iii) frozen workers form suitable samples for their quantification, (iv) they are of special practical and scientific interest. The last point was specifically true for *L. passim*, *C. mellificae*, and *C. bombi*, whose distribution in Italy is largely understudied.

## 2. Materials and Methods

### 2.1. Sampling

**Figure 1:**
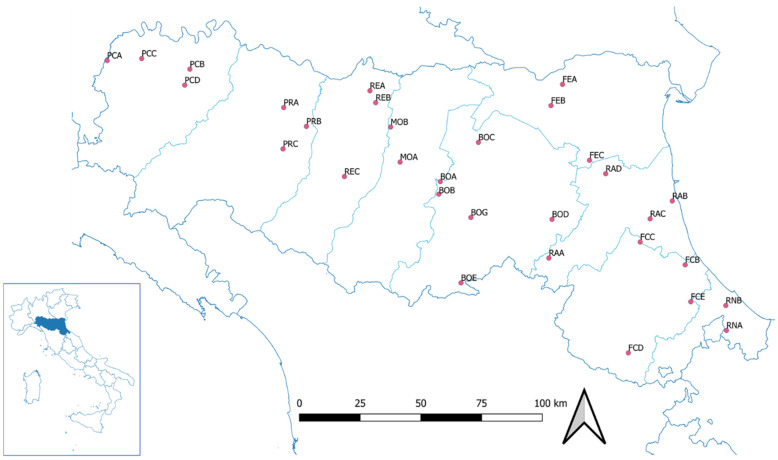
Geographical localization of the investigated apiaries in the Emilia–Romagna region.

Thirty-one apiaries distributed in all provinces of the Emilia–Romagna region were investigated (Figure 1). The apiaries were named after the province code, to which a progressive letter was added (Table 1).

In the study year (2021), the same three colonies were sampled four times for each investigated apiary, namely in April, June, September, and November. Four apiaries (BOC, FCD, RNA, and RNB) missed the first sampling, as the respective owners joined the monitoring plan later.

For each investigated colony, approximately twenty-five worker bees were sampled from the external combs [57], on which older and, therefore, more likely to be infected workers tend to congregate [58,59]. All the samples were stored at −80 °C until analysis.

### 2.2. Extraction of Nucleic Acids

Each sample was processed as a pool. Ten bees were placed in a 2mL microtube with 300 µL of DNA/RNA Shield (Zymo Research, Irvine, CA, USA) and crushed with a TissueLyser II (Qiagen, Hilden, Germany) for 3 min at 30 Hz, as previously reported [60,61]. The obtained suspension was split into two aliquots, from which DNA and RNA were separately extracted. The extraction of the nucleic acids was performed with the Quick DNA Microprep Plus Kit (Zymo Research) and Quick RNA Microprep Plus Kit (Zymo Research). During the process, the modified manufacturer’s instructions for solid tissue processing were followed [19,62]. The obtained nucleic acids were eluted in 100 µL of DNAase-RNase-free water and the extracts were stored at −80 °C until the qPCR assays.

### 2.3. Quantitative Real-Time PCR Assays

The extracted DNA and RNA were used in a Real-Time PCR (qPCR) assay to quantify the abundance of each pathogen in the samples. The DNA was used to analyse *N. ceranae* and trypanosomatids, whereas RNA was used for the viruses. The qPCRs were performed using the primers reported in Table 2.

For each target gene, a total reaction volume of 25 µL was prepared as previously described [57,62] using SYBR™ green assays with forward and reverse primers (2 µM), and 3 µL of nucleic acid extract. For the DNA and the RNA, the SYBR PowerUp™ SYBR™ Green Master Mix (ThermoFisher, Waltham, MA, USA) and the Power SYBR™ Green Cells-to-CT™ Kit (ThermoFisher Scientific) were used, respectively. The qPCRs were performed on QuantStudio™ 3 Real-Time PCR System (ThermoFisher Scientific), following the protocols for either gene sequence [63,64,65,66,67]. DNA and RNA, previously extracted from positive honey bee samples, were used as positive controls. Sterile water was used as a negative control in all analytical steps. All the analyses were conducted in duplicate.

For each target gene, a standard curve was generated by amplifying serially diluted recombinant plasmids containing the pathogen-specific DNA fragment from 1 × 10^1^ to 1 × 10^9^ copies in a qPCR assay on QuantStudio™ 3 Real-Time PCR System (ThermoFisher Scientific), as previously reported [19,55,57,62], following the amplification and quantification protocols [63,64,65,66,67].

### 2.4. Statistical Analysis

The values of pathogens abundance used for statistical analysis referred to the pool of 10 bees for each investigated colony. For each sample, the pathogen abundance was determined by averaging the two technical replicates of each PCR assay. For each apiary, the pathogen abundance was calculated by averaging the data obtained from the three investigated colonies. 

A principal component analysis (PCA) was conducted to explore similarities and possible clusters of apiaries according to a regional longitudinal gradient, using the mean number of pathogen copies as a variable. Calculations were carried out over the whole sampling year. After the PCA, the correlation strength between each pathogen’s abundance and longitude value was tested with a Pearson correlation test. 

Before proceeding with further analysis, the assumption of normal distribution of errors was checked with Shapiro–Wilk test. As the normality test was failed, a non-parametric approach was followed.

To test the effect of the month of sampling on each pathogen’s abundance, Kruskal–Wallis tests were used. When the last was found to be significant, Dunn’s test was applied as a post-hoc pairwise comparison. The apiary average prevalence of each pathogen was also calculated per month as a percentage ratio between the positive and total samples. A Chi-square test pairwise comparison with Yates’ correction was then applied to investigate the pathogen prevalence among the sampling months.

For all statistical tests, a significance threshold at α = 0.05 was assumed.

Pathogens whose presence was not detected in any of the samples by real-time PCR were excluded from analyses.

All the statistics were calculated with R studio version 4.1.2. using packages *FactoMineR* and *ggplot2*.

## 3. Results

In total, 360 samples were analyzed. None of the samples were found to be positive for any of the three trypanosomatid species. The highest prevalence, considering the four sampling points, was found for DWV (56.0%), followed by CBPV (48.5%), *N. ceranae* (42.9%) and ABPV (7.3%). Only three colonies were found to be positive for KBV infection (0.8%) (Table S1).

**Figure 2:**
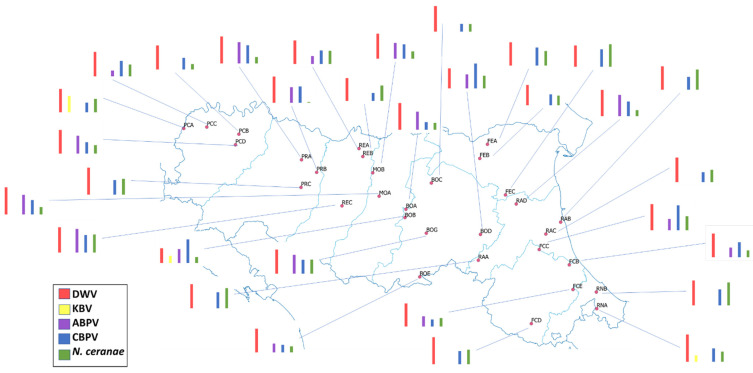
Pathogen abundance in the apiaries under the study. Bars represent the mean abundance of DWV, KBV, ABPV, CBPV and *N. ceranae* in the investigated apiary (Detailed valuas are reported in Table S2).

Concerning abundance, the highest mean number of pathogen copies was found for DWV and CBPV (1.36 × 10^10^ and 7.60 × 10^10^, respectively). Lower values were detected for ABPV (1.17 × 10^6^), *N. ceranae* (2.14 × 10^5^) and KBV (1.34 × 10^3^) (Figure 2 and Table S1).

### 3.1. Geographical Distribution

**Figure 3:**
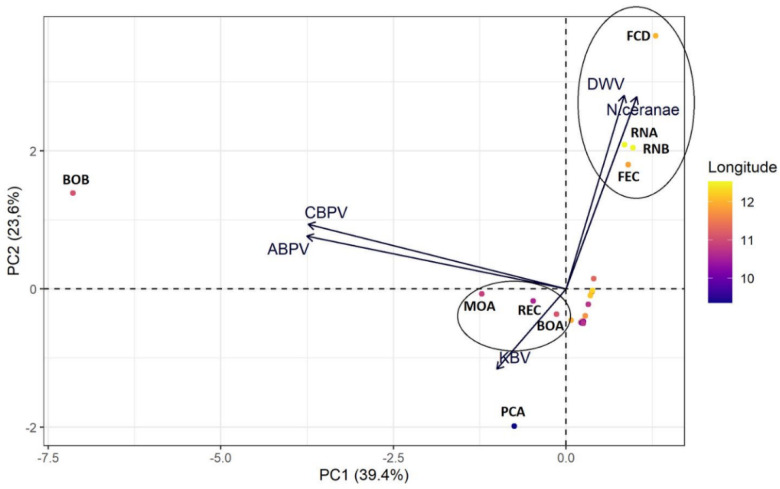
Graphical interpretation (biplot) of principal component analysis (PCA) on the abundance values of the considered pathogens for each apiary. The longitude is shown by different colours: bright and dark shades correspond, respectively, to apiaries closer to the coast (east) and the inland (west).

In the PCA, two components from the pathogen abundance were chosen at each apiary. The first one (PC1) was positively correlated with DWV and N. ceranae. The second one (PC2) was positively correlated with these pathogens, as well as with ABPV and CBPV (Figure 3 and Table 3).

The principal component analysis showed one cluster along the second principal component, corresponding to a group of easternmost apiaries (FCD, RNA, RNB, FEC), whereas the other apiaries were assembled closer to the chart origin. Three apiaries (MOA, REC and BOA) located in central areas of the region clustered following the second principal component. The BOB and PCA apiaries, located in the central and western part of the region, respectively, occupied the marginal positions in the biplot.

There was a positive correlation for *N. ceranae* (t = 2.192, *p*-value = 0.0365, cor = 0.377) and longitude value. No significant correlation was found for DWV (t = 1.805, *p*-value = 0.081, cor = 0.318), ABPV (t = −0.477, *p*-value = 0.637, cor = −0.088), and CBPV (t = −0.165, *p*-value = 0.8715, cor = −0.031). No correlation was performed for KBV, because only three apiaries were found to be positive in the total sampling year.

### 3.2. Seasonal Trend

**Figure 4:**
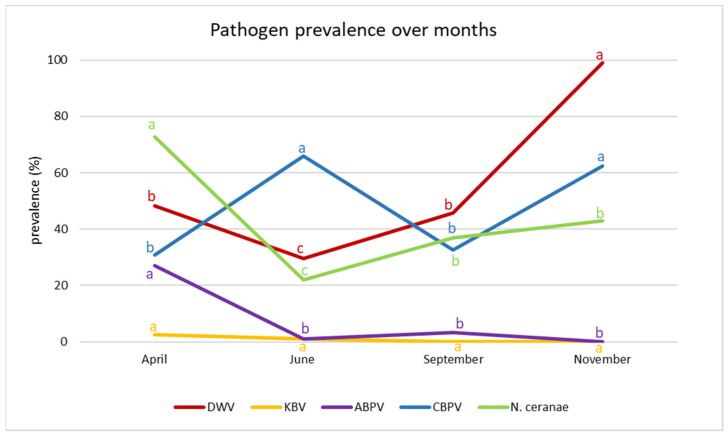
Pathogen prevalence over months. Different letters denote significant differences in the prevalence of individual pathogens.

The prevalence of the considered pathogens showed a distinctive seasonal trend (Figure 4). DWV and *N. ceranae* were most prevalent in April (48.2% and 72.8%, respectively), while their prevalence decreased in June and rose in September and November. On the other hand, ABPV decreased over the months. The prevalence of DWV increased over time, with a peak in November (98.9%). CBPV showed a fluctuating trend, with significant increases in June (65.9%) and November (62.4%). The prevalence of KBV was very low throughout the year and did not significantly fluctuate over time (Figure 4).

The results of the Kruskall–Wallis tests conducted on the abundance data of each pathogen are reported in Table 4. Except for KBV, the sampling month resulted in a significant predicting variable.

**Figure 5:**
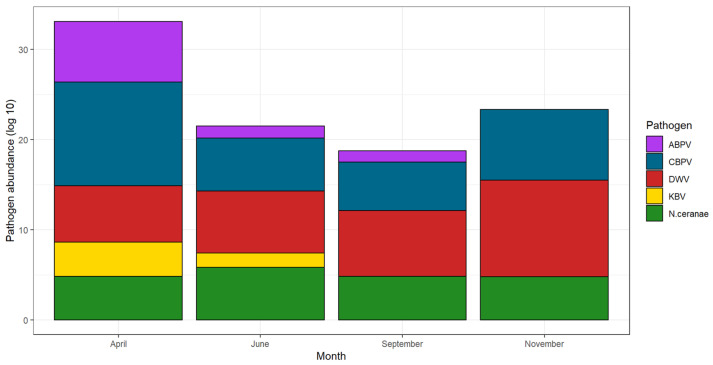
Cumulative pathogen abundance over the months. For a better visualization, data are shown as decimal logarithms.

Total pathogen abundance was higher in April and November (Figure 5).

**Figure 6:**
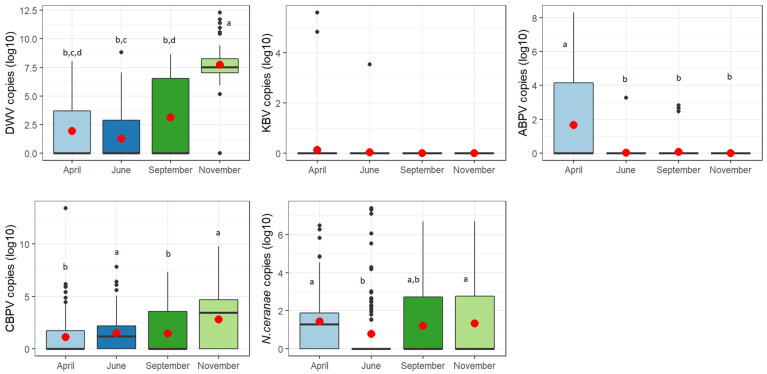
Abundance of each considered pathogen over the year. For a better visualization, data are shown as decimal logarithms. Means are indicated by red dots. Different letters denote significant differences between months.

The results of the post-hoc tests (Supplementary Table S3) revealed that the majority of significance in the comparisons was found between April and June and between April and November. ABPV and *N. ceranae* had a higher mean abundance in April, while DWV and CBPV had higher values in November (Figure 6).

## 4. Discussion

The frequent losses in honey bee colonies that occurred in recent years in Europe and other continents promoted the establishment of monitoring plans in many countries, to improve our understanding of the underlying factors and adopt the needed countermeasures. This study is part of BeeNet, an Italian multiannual monitoring plan that is conducted nationwide. Despite the large scale of the study, we presented results obtained in apiaries situated in the Emilia Romagna region. We focused on a range of pathogens that were reputed to be implicated in the colony losses [1] detected in the first year of the monitoring action (2021). Five out of eight of the considered pathogens were found to infect the examined apiaries, namely, the viruses DWV, CBPV, ABPV, and KBV, and the microsporidium *N. ceranae*.

In general, DWV was the most prevalent pathogen, following a previous study carried out in Italy in 2009–2010 where the prevalence value was higher (68%) [2]. A similar prevalence value of DWV-A was recorded in the Veneto region in 2020 [56]. DWV was reported as the most prevalent virus in Europe [68] and the other continents [27]. CBPV was the second most prevalent pathogen, occurring in almost half of the analyzed honey bee samples. The higher prevalence values of this virus (82.2–98.8%) were recently found in Veneto [56]. Since, in 2009–2010, the prevalence of CBPV in Italy was only 8% [2], this study confirms the exponential increase in the incidence of this virus observed over the last decade, as in other countries, including the UK [42], USA [69], and China [70]. ABPV and KBV were the least prevalent viruses in Emilia Romagna, especially KBV, which was only detected in three hives (0.8%). KBV was first identified in Italy in 2013 [54] and has rarely been found since then [2,56], with a predominant distribution in Asia, the USA and Australia [71].

Within the non-viral pathogens, the prevalence of *N. ceranae* in Emilia Romagna was similar to overall reports for Italy in 2009–2010 [2] but lower than the prevalence recorded in other European countries, including Belgium (93%), Serbia (79–95%) and Spain (66%) [72,73,74]. The three investigated trypanosomatids were not found in any of the colonies. In the last national survey, trypanosomatids were not searched for; therefore, the only data available for comparison are from the neighbouring Veneto region, showing a high prevalence of *L. passim* (48.8–62.2%) in 2020–2021 [56]. Similar results (62.3%) were obtained in a nine-year survey for trypanosomatids, conducted in Serbia [75]. The only other Italian study investigating trypanosomatids was carried out on honey samples supplied by beekeepers in Northern Italy. A total prevalence of *L. passim* of 78% was found in honey, with Friuli-Venezia Giulia, Emilia-Romagna and Trentino-Alto Adige scoring the highest values [76]. Data on both the presence and distribution of *C. mellificae* and *C. bombi* are even more scattered. *C. mellificae* was detected in honey bees for the first time in Italy in 2010, in the Latium region [77]; however, after the discovery of *L. passim*, the sequence was attributed to this new trypanosomatid. In 2020, *C. mellificae* was found in one single hive in the Veneto region [56]. The presence of trypanosomatids in honey bees is still poorly monitored both in Italy and elsewhere, although they are recognized as among the most prevalent bee parasites, contributing to the increased colony mortality in Europe and the USA [44]. However, our poor understanding of the epidemiologic details of trypanosomatid infections means that comparisons among the results obtained over different years and locations are excessively speculative. This is even more true when the results refer to dissimilar kinds of samples (e.g., worker bees from individual colonies and bulks of honey produced by different hives). Coinfections between *L. passim* and *N. ceranae* [75,78] were not confirmed by the present study.

Concerning the abundance of detected pathogens, the highest loads were found for CBPV and DWV (7.60 × 10^10^ and 1.36 × 10^10^ copies, respectively). A viral load higher than 10^10^ pathogen copies is generally related to the presence of disease symptoms for both viruses [29,30,79]. Therefore, the analyzed bees belonging to the monitored colonies did not present any symptoms related to both viral infections. It should be noted that the virulence and subsequent symptomatology of these viruses can vary depending on the means of transmission and the presence of co-infections/infestations [80,81]. ABPV, *N. ceranae* and KBV were found to have a lower abundance. Given the higher virulence of ABPV and KBV [31,80], apiaries with detected viral loads of around 10^6^ should be kept under control.

The geographical distribution of pathogens in the Emilia–Romagna regions highlighted a correlation between the DWV and *N. ceranae* infection in the East part (Romagna) compared to the west of the regions (Emilia). These differences could be related to the different health management of apiaries by beekeepers, probably due to different traditions or customs. *N. ceranae* and *V. destructor* (the main vector of DWV) originate from Asian countries and can replicate in a warm environment [17,82]. The apiary in the east Emilia–Romagna has a closer proximity to the sea, which contributes to a more suitable environment for *N. ceranae* and DWV infections [83,84,85,86,87]. Additionally, in 2021, a higher average temperature was recorded in Romagna (17 °C) than in Emilia (14 °C), creating a more suitable environment for these organisms (https://simc.arpae.it/dext3r/ (accessed on 13 April 2021)). In the global view of climatic change, this may lead to an increase in cases of exotic pathogens [10,88,89].

In this investigation, the seasonality of pathogen occurrence followed different specific trends for each pathogen. Usually, the prevalence and the abundance of pathogens are strictly related to the bee population dynamics, especially to brood cycles [7,10,14,90].

DWV and ABPV transmission is also linked to *V. destructor* [26,28]. Their rate of prevalence, abundance and virulence followed the same trends of mite infestation [69,91,92,93]. The high infection of ABPV and DWW in April may be linked to a constant infection that remains active during the winter. This leads honey bees being infected in the new brood cycle in the spring [94,95,96]. The slight decrease in terms of prevalence and abundance in June could be in line with the increase in bee population, diluting the pathogens among more hosts [97]. The proliferation of both *V. destructor* and viruses cannot be limited by acaricide treatments. Although mite infestations can successfully be reduced, the virus can persist inside the hosts at low prevalence and loads [10]. In this case, the ABPV cycles decrease over the season, and the summer acaricide treatments seem to be effective at inhibiting its spread within the bee population, as previously reported [94,96]. On the other hand, the summer treatments did not affect the persistence and circulation of DWV, since its infection increased after the summer. Instead, the winter reductions in population and the winter acaricide treatments seem to be useful to limit the damage caused by higher rates of DWV infection.

In recent studies, the CBPV was considered to be strictly linked to spring and related to colony developmentl however, cases of this virus were detected in all beekeeping seasons and during the winter [38,98]. The analyzed CBPV trends confirmed the importance of the re-emerging disease [42]. The virus was present in all seasons in the investigated apiary, as previousy reported [38,41,99]. The higher abundance in April and September reflected the higher prevalence in June and November. These findings could be related to the first and classic aetiology of the disease, where a higher number of cases were found in late spring/early summer [38,39]. The absence of a temporal pattern may be associated with the oral transmission of CBPV without a vector, or due to the genetic diversity of the virus [38,98].

Due to the very limited KBV-positive colony detected in this study, its seasonal dynamics cannot be analyzed. This disease is not present in Italy, except for random findings in some bee colonies in the Latium region and *V. velutina* sampled in the Liguria region [54,55].

*N. ceranae* is the most diffused and most frequent microsporidia detected in honey bee colonies worldwide [1,100]. Previous surveys in Europe highlighted the differences and fluctuations in *N. ceranae* prevalence and abundance depending on the year and the geographical localization of the apiary [101]. In this investigation, its prevalence and abundance were detected in all samples, confirming the lack of seasonality that was previously reported [102,103]. The highest incidence was observed during the colony development in April and in November before the wintering, in line with the life cycles of the microsporidia [104], as previously reported in other countries [100,105,106,107,108,109].

## 5. Conclusions

This investigation focused on the health of managed honey bees, confirming the important role of monitoring surveys on bee diseases. The impact of management practices on bee colonies leads to an intensification of these surveys, not only because the impact of the diseases causes direct damage to beekeeping, but also because the circulation of these pathogens may affect other pollinators [110]. The health of the honey bee is strictly connected to the health of pollinators, and vice versa. A change in perspective is necessary, considering a One-Health Approach to honey bee diseases [111,112,113].

Further studies are needed to better understand the health status of Italian and European honey bee colonies to prevent the diffusion of diseases and to protect the environment.

## Figures and Tables

**Table 1:** Geographical characteristics of the investigated apiaries (a.s.l.= above sea level).

Apiary Code	Province	Municipality	a.s.l.
BOA	Bologna	Valsamoggia	60 m
BOB	Bologna	Valsamoggia	114 m
BOC	Bologna	Sala Bolognese	13 m
BOD	Bologna	Dozza	89 m
BOE	Bologna	Monghidoro	582 m
BOG	Bologna	Sasso Marconi	155 m
FCB	Forlì-Cesena	Cesena	9 m
FCC	Forlì-Cesena	Forlì	8 m
FCD	Forlì-Cesena	Bagno di Romagna	679 m
FCE	Forlì-Cesena	Borghi	63 m
FEA	Ferrara	Ferrara	7 m
FEB	Ferrara	Ferrara	8 m
FEC	Ferrara	Argenta	8 m
MOA	Modena	Formigine	81 m
MOB	Modena	Campogalliano	26 m
PCA	Piacenza	Ziano Piacentino	298 m
PCB	Piacenza	Pontenure	78 m
PCC	Piacenza	Gragnano Trebbiense	88 m
PCD	Piacenza	San Giorgio Piacentino	323 m
PRA	Parma	Parma	50 m
PRB	Parma	Montechiarugolo	79 m
PRC	Parma	Langhirano	298 m
RAA	Ravenna	Casola Valsenio	204 m
RAB	Ravenna	Ravenna	7 m
RAC	Ravenna	Ravenna	8 m
RAD	Ravenna	Lugo	8 m
REA	Reggio Emilia	Novellara	16 m
REB	Reggio Emilia	Campagnola Emilia	19 m
REC	Reggio Emilia	Viano	433 m
RNA	Rimini	Montescudo	209 m
RNB	Rimini	Rimini	46 m

**Table 2:** List of the primers used to analyse the pathogens in this study.

Target	Primer Name	Sequence (3′-5′)	Reference
*N. ceranae*	Hsp70_F	GGGATTACAAGTGCTTAGAGTGATT	[63]
	Hsp70_R	TGTCAAGCCCATAAGCAAGTG	
*C. mellificae*	Cmel_Cyt_b_F	TAAATTCACTACCTCAAATTCAATAACATAATCAT	[64]
	Cmel_Cyt_b_R	ATTTATTGTTGTAATCGGTTTTATTGGATATGT	
*L. passim*	Lp_Cyt_b_F	CGAGCTCATAAAATAATGTAAGCAAAATAAG	[64]
	Lp_Cyt_b_R	TTTTAGCAATATTTTAGCAACAGTACCAG	
*C. bombi*	Cbom_119Fw	CCAACGGTGAGCCGCATTCAGT	[65]
	Cbom_119Rv	CGCGTGTCGCCCAGAACATTGA	
KBV	KBV 83F	ACCAGGAAGTATTCCCATGGTAAG	[66]
	KBV 161R	TGGAGCTATGGTTCCGTTCAG	
DWV	DWV Fw 8450	TGGCATGCCTTGTTCACCGT	[67]
	DWV Rev 8953	CGTGCAGCTCGATAGGATGCCA	
ABPV	APV 95F	TCCTATATCGACGACGAAAGACAA	[66]
	APV 159R	GCGCTTTAATTCCATCCAATTGA	
CBPV	CPV 304F 79	TCTGGCTCTGTCTTCGCAAA	[66]
	CPV 371R	GATACCGTCGTCACCCTCATG	

**Legend**. KBV: Kashmir bee virus; DWV: deformed wing virus; ABPV: acute bee paralysis virus; CBPV: chronic bee paralysis virus.

**Table 3:** Relationship between Principal Components (PC1 and PC2) and pathogen abundance variables.

Pathogen	PC1	PC2
DWV	0.152	0.653
KBV	−0.181	−0.2716
ABPV	−0.676	0.178
CBPV	−0.673	0.219
*N. ceranae*	0.184	0.649
Eigenvalues	1.971	1.178
Variance explained	39.43%	23.55%

**Table 4:** Results of Kruskal–Wallis tests of pathogen abundance in the sampling months. Significant values are shown in bold.

Pathogen	Test Value	*df*	*p*-Value
DWV	186.16	3	**0.000**
KBV	4.2262	3	0.238
ABPV	63.556	3	**0.000**
CBPV	31.852	3	**0.000**
*N. ceranae*	21.312	3	**0.000**

## Data Availability

Not applicable.

## Supplementary Materials

The following supporting information can be downloaded at: https://www.mdpi.com/article/10.3390/vetsci9080437/s1, Table S1. Mean number of copies for investigated apiary in the Emilia-Romagna region during April, June, September, and November 2021. Table S2. The mean annual abundance of DWV, KBV, ABPV, CBPV and N. ceranae in the investigated apiary. Table S3. *p* values resulting from the post-hoc Dunn’s test for pairwise comparisons (ns = non-significant).
