# Supplementary material for: The Epidemiological Situation of the Managed Honey Bee (Apis mellifera) Colonies in the Italian Region Emilia-Romagna
Source: Vet Sci. 2022 Aug 17;9(8):437. doi: 10.3390/vetsci9080437 (PMC9412502; doi:10.3390/vetsci9080437)
Supplement: Supplementary file 1 [file vetsci-09-00437-s001.zip › Supplementary Table S2.pdf]

Supplementary Table S2. The mean annual abundance of DWV, KBV, ABPV, CBPV and *N. ceranae* in the investigated apiary.

**Apiary**	DWV copies	KBV copies	ABPV copies	CBPV copies	*N. ceranae* copies
BOA	2.38E+09	0.00E+00	2.10E+06	4.79E+02	2.84E+02
BOB	5.22E+07	5.67E+03	1.86E+07	2.26E+12	1.36E+03
BOC	5.59E+10	0.00E+00	0.00E+00	1.85E+03	1.66E+03
BOD	1.04E+07	0.00E+00	7.99E+04	5.13E+08	2.87E+04
BOE	1.77E+08	0.00E+00	1.10E+03	3.92E+02	1.18E+02
BOG	5.20E+06	0.00E+00	1.82E+05	7.91E+03	8.28E+03
FCB	2.19E+08	0.00E+00	2.29E+03	1.74E+05	2.40E+02
FCC	2.09E+07	0.00E+00	1.54E+03	9.30E+06	8.54E+03
FCD	2.25E+11	0.00E+00	0.00E+00	3.42E+05	1.39E+06
FCE	1.01E+08	0.00E+00	3.56E+03	2.79E+02	8.30E+02
FEA	1.06E+08	0.00E+00	0.00E+00	1.82E+06	9.75E+04
FEB	2.35E+06	0.00E+00	0.00E+00	2.53E+03	1.03E+03
FEC	8.56E+05	0.00E+00	0.00E+00	7.25E+04	2.06E+06
MOA	3.17E+09	0.00E+00	7.88E+06	1.22E+05	4.55E+02
MOB	1.00E+07	0.00E+00	2.70E+04	1.07E+04	1.15E+02
PCA	3.12E+06	3.38E+04	0.00E+00	4.87E+02	5.58E+03
PCB	2.36E+08	0.00E+00	0.00E+00	1.14E+04	7.24E+01
PCC	6.29E+06	0.00E+00	3.83E+01	2.08E+04	1.88E+03
PCD	3.91E+06	0.00E+00	9.08E+04	1.46E+03	2.20E+02
PRA	3.54E+07	0.00E+00	9.20E+05	1.25E+05	6.55E+01
PRB	2.01E+07	0.00E+00	2.24E+04	3.41E+04	1.60E+00
PRC	3.19E+07	0.00E+00	0.00E+00	1.14E+04	2.49E+04
RAA	5.67E+06	0.00E+00	0.00E+00	2.98E+04	4.12E+05
RAB	3.62E+06	0.00E+00	0.00E+00	3.94E+03	4.29E+05
RAC	2.01E+10	0.00E+00	0.00E+00	1.47E+04	1.54E+05
RAD	2.22E+07	0.00E+00	9.23E+05	1.21E+04	4.68E+01
REA	3.54E+06	0.00E+00	1.55E+02	5.75E+03	4.49E+03
REB	9.35E+07	0.00E+00	0.00E+00	5.87E+02	2.46E+05
REC	1.24E+07	0.00E+00	4.04E+06	8.07E+04	1.12E+05
RNA	2.23E+11	3.85E+02	0.00E+00	3.57E+05	1.59E+04
RNB	8.21E+06	0.00E+00	0.00E+00	2.83E+04	2.28E+06
