# Supplementary material for: The Epidemiological Situation of the Managed Honey Bee (Apis mellifera) Colonies in the Italian Region Emilia-Romagna
Source: Vet Sci. 2022 Aug 17;9(8):437. doi: 10.3390/vetsci9080437 (PMC9412502; doi:10.3390/vetsci9080437)
Supplement: Supplementary file 1 [file vetsci-09-00437-s001.zip › Supplementary Table S3.pdf]

Supplementary Table S3. P values resulting from the post-hoc Dunn's test for pairwise comparisons (ns = non-significant).

Pathogen	April-June	April-November	April-September	June-November	June-September	September- November
DWV	ns	0.000	ns	0.000	0.000	0.000
ABPV	0.000	0.000	0.000	ns	ns	ns
CBPV	0.004	0.000	ns	ns	ns	0.000
N. ceranae	0.000	ns	ns	0.001	ns	ns
